# O.5.2-5 Piloting a text message intervention to increase physical activity and exercise after post-stroke rehabilitation: the KATS study (Keeping Active with Texting after Stroke)

**DOI:** 10.1093/eurpub/ckad133.246

**Published:** 2023-09-11

**Authors:** Jacqui Morris, Linda Irvine, Albert Farre, Stephan Dombrowski, Jenna Breckenridge, Gozde Ozakinci, Therese Lebedis

**Affiliations:** University of Dundee, UK; University of Dundee, UK; University of Dundee, UK; University of New Brunswick, Canada; University of Dundee, UK; University of Stirling, UK; NHS Grampian, UK; j.y.morris@dundee.ac.uk

## Abstract

**Purpose:**

After stroke rehabilitation, people feel abandoned by services and struggle to undertake physical activities to support recovery and health. Text messaging interventions can reach many people at low cost but have not been used widely after stroke. This study pilot-tested a text message intervention (KATS) to promote post-rehabilitation activity.

**Methods:**

We developed KATS using: formative research on post-stroke activity; stakeholder engagement (people with stroke and rehabilitation therapists) to identify priorities; the Health Action Process Approach to structure the behaviour change intervention; and stakeholder review before pre-testing. KATS components included: increasing motivation; goal setting and planning; self-monitoring; coping planning; and maintaining regular activity. KATS comprised 95 messages, delivered by computer programme, over 12 weeks. Text messages explained and modelled KATS components using Behaviour Change Techniques effective for changing health behaviours. Messages used conversational language to encourage engagement, some asked questions on current activities. Quotes from survivors modelled behaviours, providing encouragement and authenticity. Some messages were personalised to include participants’ names.

We piloted KATS with community-dwelling stroke survivors, using mid and end-of-intervention interviews to explore experiences of KATS. We analysed qualitative data using Normalisation Process Theory to examine how participants made sense of KATS and embedded it in everyday activities.

**Results:**

Following piloting, from 24 interviews with 12 participants, we derived four analytical themes: (1) Making sense of KATS: Timing and complementarity in the rehabilitation journey; (2) Engaging with KATS: Connection and identification with others; (3) Making KATS work: flexibility and tailorable guidance; (4) Appraising the worth of KATS: encouragement and friendliness. Participants differentiated KATS from current rehabilitation practice, finding it relevant, fitting, and worthwhile. They reported variations in engagement with behaviour change techniques, but participants could tailor KATS use, making it work for them in different ways.

**Conclusions:**

KATS was seen as useful and acceptable to stroke survivors. Perceived benefits went beyond promoting physical activity, to feeling supported and connected. Future research will test effectivenes of KATS in promoting physical activities and explore associations with functional, social and emotional secondary outcomes. The study illustrates the importance of co-design in interventions for clinical populations.

**Funder:**

Scottish Government Chief Scientist Office.

